# Women's experiences of sexual harassment in the retail clothing industry: a grounded theory study

**DOI:** 10.3389/fpsyg.2024.1374188

**Published:** 2024-06-04

**Authors:** Maryam Akbari, Mohammad Hossein Kaveh, Rosanna Cousins

**Affiliations:** ^1^Department of Health Promotion, School of Health, Shiraz University of Medical Sciences, Shiraz, Iran; ^2^Department of Psychology, Liverpool Hope University, Liverpool, United Kingdom

**Keywords:** sexual harassment, coping responses, female employees, grounded theory, silence, shame

## Abstract

**Introduction:**

Sexual harassment is a significant problem in workplaces all over the world. Women's reactions to sexual harassment are influenced by various factors. The aim of the current study was to investigate how women respond sexual harassment in the retail clothing industry.

**Methods:**

In-depth face-to-face interviews were conducted with 16 women aged 23–44 years (mean 29.18 years) employed for at least 3 years in clothes shops in Shiraz, Iran. A grounded theory approach was used to analysis the data and raise hypotheses.

**Results:**

The main perpetrators of sexual harassment for female saleswomen were male customers. The women experienced conflict-induced stimulation (core phenomenon) when they were faced with sexual harassing behaviors (causal conditions). Such stimulation prompted three types of coping strategies: silence, avoidance, or confrontation. Intervening factors like the characteristics of the Iranian society (including family mores, state-imposed hijab regulations, patriarchal culture, educational system, and regulatory monitoring) and contextual factors (including individual and environmental factors and particularly employer expectations) were found to influence the selection of strategies used as well as their potential consequences in challenging situations.

**Conclusion:**

The current study used a grounded theory approach to produce an explanatory storyline that can be tested. Sexual harassment induces conflict-induced stimulation and responses are influenced by intervening conditions, contextual factors, selected strategies, and the perceived consequences of the response. The findings of the grounded theory study suggest that there are negative consequences, particularly in terms of lack of employer support and losing one's job, shame, and family disapproval which act as barriers for female saleswomen to counteracting sexual harassment from male customers. Such an understanding can also be applied to develop educational policies to support women as well as ameliorate the prevalence of this essentially illegal problem.

## 1 Introduction

Sexual harassment in workplaces is prohibited according to the Civil Rights Act of 1964 (Hersch, 2018). Although nine out of every ten countries in the world have adopted regulations against sexual harassment in workplaces (Bondestam and Lundqvist, 2020), it is still a pervasive and prevalent phenomenon across the globe (Zeigler-Hill et al., 2016; Nielsen et al., 2017). Challenging reasons for the longevity of sexual harassment into the 2020s, despite protective laws and policies regarding the deed (sexual harassment), the context (sexual discrimination), and protest movements (e.g., the #Me Too movement), can be found in the seminal work of MacKinnon (1979). MacKinnon argued that sexual harassment is seen because of segregation in the organization of work which maintains the long-standing unequal power and control between men and women. Critically, men have a superior status (evidenced most clearly in pay differences); and women were most useful in roles in which they can be exploited sexually for economic benefit (MacKinnon, 1979). Thus, some sectors employ women extensively, if not exclusively, because of certain required characteristics. That is, work in which a woman can please men by fulfilling some sex-role expectations is assimilated into the job role. More than 50 years later, the vulnerability of women in normative sex-related employment opportunities persists in many sectors. In this way women can be exploited economically in terms of working conditions and expectations of behavior (Good and Cooper, 2016; Zhao and Ghiselli, 2016). Ultimately, a precondition for answering the question at the close of Cortina and Areguin (2021)'s review of sexual harassment in the workplace: “How do we combat the many slights and indignities that combine to relegate women … to the margins of organizational life?” (p. 304) is to understand women's experiences of sexual harassment in workplaces in detail. A new approach to combating sexual harassment is dependent on an up-to-date interpretation of women's responses to encounters of sexual harassment that includes investigation of *why* they behave as they do. That is the principle aim of this research.

The International Labour Organization (2011) distinguished five forms of sexual harassment including undesirable physical touch with sexual intentions (e.g., kissing, patting), verbal harassment (e.g., unpleasant conversations about private lives or body organs, obscene jokes), gestural harassment (e.g. suggestive body language, sexual gestures using fingers), written or visual harassment (e.g., sending or displaying pornographic materials, sexually explicit images), and emotional/mental harassment (e.g., persistent or unpleasant requests, unwanted invitations). In addition to these generally observable behaviors that can be recognized as explicit sexual harassment, Cortina and Areguin (2021) point out that there are many sexist insults and crude comments that are also sexually harassing acts whose purpose is to denigrate women. Altogether, sexual harassment in workplaces brings about various consequences, and it has been considered among the most important challenges in human resources management (Kahsay et al., 2020). Sexual harassment can result in multiple negative consequences that involve an individual's health and their professional performances (Zeigler-Hill et al., 2016; Perumalswami and Jagsi, 2020). Physical problems like headache, nausea, weight loss, and physical issues (Fitzgerald et al., 1988; Kaltiala-Heino et al., 2018) and psychological consequences like depression, anxiety, and stress have been reported by the women experiencing sexual harassment (Wolff et al., 2017). Regarding the organizational dimension, sexual harassment can lead to lower job satisfaction, reduction of organizational participation, and career change (Chan et al., 2008; Zeigler-Hill et al., 2016; McLaughlin et al., 2017).

The early literature investigating sexual harassment in employees found that participants who were asked questions revolving around “what would they do if harassed” typically reported that they would tell the harasser to stop, but in practice, confronting a harasser, even verbally to tell them to stop, was not the approach of victims (Gutek and Koss, 1993). A US survey conducted in the wake of the Harvey Weinstein sexual abuse allegations (October 2017) and the re-emergence of the #Me Too movement confirmed that the scale of sexual harassment and assault, particularly against women, poses a substantial problem (Stop Street Harassment, 2018). The majority (81%) of the 1,000 women and approaching half (43%) of the 1,000 men who participated in that survey reported that they had experienced some form of sexual harassment and/or sexual assault—and many multiple times. This phenomenon has been reported in many other countries besides the US. For example, a large-scale national cohort survey to assess the extent of workplace sexual harassment in Iceland (*N* = 30, 4,003) found that that one in three females (33.5%) reported that they had been harassed, with young, educated women having the highest rates of exposure (Jonsdottir et al., 2022). Other survey studies have also confirmed that there are high levels of workplace sexual harassment, that occurs in a range of workplaces, and that the perpetrators are not only fellow workers, but also clients. For example, Notaro et al. (2021) surveyed a sample of 330 healthcare professionals, and found that the majority (83%) had experienced instances of sexual harassment or attack by patients.

Whilst the literature asserts that workplace sexual harassment is common across the globe, it is also apparent that the phenomenon has rarely been investigated in Iran. Although sexual harassment is a reality in Iran, traditional limitations and some cultural considerations prevent women from reporting it, and thus a significant proportion of the issue is hidden (Mesri et al., 2021). Additionally, because very few studies have been conducted in this field in Iran, planning evidence-based interventional programs to eliminate, or at least ameliorate this concealed problem is a challenge. Thus, building on observations that sexual harassment thrives in places of work where men lead and women are both less in number and status (Dobbin and Kalev, 2017), and that training and reporting initiatives have not led to any meaningful reduction in the levels of sexual harassment (Cortina and Areguin, 2021), there remains a need for meticulous investigation to describe and understand the causes and consequences of sexual harassment to design effective solutions that will work. Women's own perceptions of the various types harassment they may experience, their reactions to harassment when it happens, and awareness of the factors that influence those reactions, according to each workplace's context and culture, can offer distinct insights to supporting interventions. Qualitative studies can provide more in-depth information, and grounded theory research that draws upon a dynamic analysis of data that contributes to data collection and ultimately building theory (Corbin and Strauss, 1990) provides an approach to describe responses to sexual harassment and its causes. Thus, the aim of the current study was to investigate women's reactions to workplace sexual harassment in Shiraz, Iran, using a grounded theory approach, and a sample of women working in small-to-medium sized independent retail clothing stores in Iran. Employees in these stores are typically employed on a short-term contract, and renewal largely depends on making enough sales. This, in turn, is dependent on a personal approach and a high level of attractiveness and conversation skills is expected by both employer and customer. Due to these conditions of employment, retail sales assistants were recognized as an appropriate population for investigating *how* and *why* women respond when confronted by sexual harassment at work.

## 2 Method

### 2.1 Study design

The qualitative study used a grounded theory research design and followed Consolidated Criteria for Reporting Qualitative Research (COREQ). Grounded theory is used to discover relevant conditions for a phenomenon, and to investigate how people in those conditions respond to the conditions alongside the perceived consequences of that response (Corbin and Strauss, 1990). Thus, this methodology provided an appropriate way to investigate responses to sexual harassment as dynamic and relatively unexplored social incidents, and to create testable theories and models from the data (Strauss and Corbin, 1998; Glaser and Strauss, 2017). The collected data were analyzed using the interrelated processes method recommended by Corbin and Strauss (1990).

### 2.2 Recruitment, participants and ethical review

The study protocol was approved by the Ethics Committee of Shiraz University of Medical Sciences, Shiraz, Iran (IR.SUMS.REC.1398.1366). The study was carried out in accordance with the Declaration of Helsinki. The study was advertised using a leaflet drop in small-to-medium sized independent retail clothing stores in Shiraz in 2020. There are usually 5–10 employees in these stores, all typically employed on a short-term contract. Renewal of a contract largely depends on success in attracting customers and making a sale. Due to these conditions of employment, retail sales assistants are vulnerable to exploitation and harassment. It was recognized that this was an appropriate population for investigating what women do when confronted by sexual harassment in the workplace. The leaflets advertising the study included the email address of first author (MA) toward registering an interest in participating in the study. Respondents were sent a full Participant Information Sheet which fully explained the interview procedure, assurances of confidentiality and anonymization of data provided. It also reiterated the inclusion criteria on the leaflets: more than 1-year working as a full-time clothing retail sales assistant, and experience of sexual harassment whilst working in the shop. Using this purposeful recruitment approach, sixteen women gave informed consent to take part in a face-to-face unstructured interview on their experience of sexual harassment. All participants took part on a voluntary basis, away from their workplace, at a time of their convenience.

### 2.3 Data collection and analysis

Ethical considerations of the study were reiterated at the start of each interview. The study procedure and privacy considerations regarding voice recording and identities were explained to the participants. That is, all voice recording would be deleted after transcription, and all identifying data would be anonymized. Then, written informed consent to perform the interviews and take discreet field notes regarding body language were obtained. Research questions were designed during the process of data collection and analysis. Analysis began with the first bit of data provided, as it directed the next question and observation. Similarly, the first interview was transcribed word for word and checked several times to directed the next interview, and so on. This systematic and sequential process of data collection and analysis engaged three types of coding—open, axial, and selective—as part of the constant comparative method to allow emergence and verification of theory put forward by Corbin and Strauss (1990). The method involved the simultaneous collection of rich interview data (over about an hour), and interpretive analyses to develop codes, themes and categories. This involved a comparative analysis of different sections of the data in terms of similarities and differences to find meaningful concepts. Data collection paths were selected based on theoretical grounds, to develop the emerging categories, and finalize, and optimize them (Corbin and Strauss, 1990; Glaser and Strauss, 2017). The rate of the collected data decreased as the study went on, but more analyses were conducted. The process was continued until the research team were convinced that theory had emerged, the categories were saturated, more interview data would not add any new information, and thus theoretical saturation was achieved (Aldiabat and Le Navenec, 2018).

### 2.4 Data trustworthiness

All stages of the research rigorous adhered to best practice, including recruitment of this hard-to-reach sample. Data collection was performed by a competent interviewer with additional expert input from other team members; all have training and experience in qualitative research methods. The interviews were conducted by a female PhD student (MA) who has Master's and Bachelor's degrees in Social Sciences. Coding processes was independently undertaken by two team members (MA and MHK) at all points of the analyses. The obtained coding instances were compared with each other, and agreement was reached before moving on. Team reflection and debrief activities also supported data trustworthiness, particularly as decisions regarding data and theoretical saturation and choice of quotes for illustration of theory in this article were made.

## 3 Results

Details of the participant's demographics are presented in Table 1.

**Table 1:** Participant's demographic characteristics.

**Code**	**Age (years)**	**Education level achieved**	**Marital status**	**Work experience (years)**
P1	44	Higher education degree	Married	10
P2	35	High school diploma	Single	11
P3	36	High school diploma	Married	9
P4	31	High school diploma	Single	6
P5	32	High school diploma	Single	6
P6	23	High school diploma	Single	6
P7	32	Higher education degree	Single	10
P8	25	High school diploma	Married	4
P9	23	High school diploma	Single	5
P10	23	Under high school	Single	3
P11	25	Higher education degree	Single	10
P12	29	High school diploma	Single	7
P13	29	High school diploma	Single	7
P14	26	High school diploma	Single	6
P15	30	High school diploma	Married	9
P16	24	High school diploma	Single	4

The analysis of the interview data revealed several classes and subclasses of response to sexual harassment, as elaborated upon in this section. A primary finding was that the main perpetrators of sexual harassment were male customers. Conflict-induced stimulation was considered the core phenomenon associated with the causal condition—the experience of sexual harassment. The characteristics of the society in which the women worked (e.g., family, patriarchy, obligatory hijab regulations, characteristics of educational systems, and regulatory monitoring) were considered the context. Intervening conditions included individual factors of the participant (e.g., personality, financial need) and environmental factors (e.g., workplace atmosphere and policies, emotional work, and supervisors' personality traits). Response strategies reported were silence, counteracting, and avoidance. These were investigated alongside consideration of the perceived negative consequences of those strategies, which included job loss, family challenges, and risks concerning one's marriage.

### 3.1 Causal conditions

Four types of sexual harassment—verbal, physical, gestural, and psychological—were identified. Verbal harassment included taunting saleswomen and flirting with them, whereas physical harassment referred to feeling a saleswoman's body or hand while handing over money or credit card. The gestural type involved male customers' gazes and winks. The psychological type included persistent proposals to establish a relationship, friendship, or temporary marriage. Psychological harassment followed by gestural and physical harassment were the most prevalent types. Participants' statements included:

*Once a customer said that he wanted to buy something for his daughter who was the same size as me. He asked me to put on the clothes, so that he could decide. As soon as I came out of the fitting room, he started feeling my body*.

*Men come to the shop come to propose. It has become so customary that they say they want clothes for my size, then they try to feel my body. Some others, including my male colleagues, try to taunt me. Some men gaze with sly expressions on their faces or become nosey. They ask personal questions like whether I am single or married. Men who come alone without their wives try to stay as long as possible as if merely looking at me satisfies them*.

All the participants mentioned that they were harassed more by married men, while single men usually made persistent proposals to them. For instance,

*Many male customers accompany their wives and harass me with their gazes. Married men create most problems. Once on a holiday, a man and his wife came to the shop. I gave several clothes to the woman to try on in the fitting room. The man was walking up and down the shop and looking slyly at me. His behavior was very strange. Suddenly, he put a piece of paper in front of me, on which he had written his phone number*.

*Whenever a man sees that there is a saleswoman, he forces his wife to come here for shopping. He enters the shop and gazes lasciviously at me. Once his wife goes into the fitting room, his behavior becomes lewd. I have seen a lot of such men*.

### 3.2 Core phenomenon

When asked about their response to the different forms of sexual harassment encountered participants generally indicated that they were unconsciously stimulated or aroused. There were, however, a variety of reactive responses from the participants that ranged from silence, to avoidance, and counteraction. This pointed to the existence of an internal conflict in choosing the type of response or reaction, which was recognized as “conflict-induced stimulation.” For example,

*Though his wife is in the fitting room, he gazes at me and starts flirting. If I react, I would have to find another job. If I do not, I have to endure a lot of pressure. I cannot be indifferent. When I see such behaviors, I feel that I am obliged to do something. Such occurrences are common for us … and, somehow, we must cope with them. However, I really do not know what I should do. On one hand, I am scared of losing my job, and on the other hand, my family members will prohibit me from going to [work] if they become aware of such occurrences*.

*Our families always instruct us on what to wear. They tell us not to wear make-up and not to converse with strange men. However, at work, the instructions I receive are completely the opposite of what I hear at home. My employer asks me to behave in a way that attracts customers. He tells me to talk and laugh with them. He prohibits the occurrence of any tension at work, and tells me I should never argue, or make a commotion, with customers. But I know that keeping silent makes the harassers bolder. I really feel pressure and do not know what to do when I see such things*.

A deeper investigation of the participants' responses revealed a spectrum of contextual, intervening factors that influenced the level of stimulation and the accompanying conflicts, as described below.

### 3.3 Contextual conditions

Characteristics of the Iranian society—particularly family values, the patriarchal culture, obligatory hijab regulations, characteristics of the education system, and faulty regulatory monitoring—emerged as the contextual conditions that resulted in conflict-induced stimulation. The family was the major influence contributing to conflict-induced stimulation. Without exception, participants stated that their families asked them to behave solemnly with men in their workplaces and to cover their body completely, so that they would remain protected from customers' harassment. For example:

*My family members always say that girls should behave solemnly. They have repeatedly told me to behave seriously with men in my workplace, and not to smile at them, so that they may not think mistakenly. They ask me to cover my body. They also instruct me to walk away, or at least to remain silent if I see any improper behaviors*.

*My family members always emphasize that my employer should not be young or male, that I should not smile or laugh in the workplace. They only allow me to work in a place where I am in charge and the [male] employer is not present. They prohibit me from wearing close-fitting clothes. I must dress modestly*.

Obligatory hijab regulations and the patriarchal system also influenced conflict-induced stimulation. Because of the lack of freedom on how women dress in Iran (which is not restricted to the obligatory hijab), women who do not fully observe the legal dress code are seen as inclined to establish a sexual relationship. That is, any woman who do not wear their hijab properly is thought of as available for extramarital relations with men, which encourages some men to initiate sexually harassing behaviors. As noted,

*The closed atmosphere of our society is another factor. Since the government has enforced hijab regulations, men have become more avaricious. Men now grow up in a constrained way. Our society brings them up with a limited perspective. If some of my hair is outside the scarf or the button of my collar is open, they look at me as though I am ready to start relationships with them. Then, they behave as they like*.

*The wearing of a hijab is so influential here. As it is obligatory and there are constraints, many women try to disobey the regulations. Then, when some men see women whose hair is not covered by their scarves or whose clothes are strange, they interpret it as a green light for starting a relationship*.

The participants also believed that the dominant patriarchal system allowed men to act freely. In other words, men are always considered to be right in any argument against any woman. For instance,

*Our society always justifies men. When you argue with men, they are always justified, and women are seen as the guilty party. I really do not know what to do*.

*Men are always justified to behave in a harassing way. They think that women are weak, and they can act as they wish. They are not scared at all. Our society is set up in a way that prompts men to think that they can bring women under control*.

The data indicated that defective regulatory monitoring mechanisms could intensify instances of harassment. Participants emphasized that no regulatory monitoring procedures existed to support victims of harassment, or to punish the perpetrators. Moreover, they complained that women were typically accused of stimulating men on occasions that raised awareness of inappropriate behaviors. They also gave examples of court cases that had not supported women who had reported significant sexual harassment. Many of the participants said that this was a significant factor in their decisions not to make any defensive reaction against a customer who harassed them. That is, it would be ineffective, and even more damaging to themselves. For example,

*Women are always condemned if they respond to their harassing customers. The law always backs men, and you will just waste your time and energy by following up on a case of harassment experienced. If you dare to take a case to the court, the clerks there will look at you as a harasser. One of my friends who had considered taking her case to court said that nothing could be done, and that no one would support us. If there were strict regulations, men would not dare to behave in such an offensive way, and we would not suffer so much. It seems as if everything supports men*.

*I have not heard about any laws that protect women against harassing men. When the law and courts support men, speaking of the law is absurd. Even if some claim that there are laws, it is like a joke. … If it was real, we would be safe and the situation would not be like this. … If any laws exist in this regard, women are implicated by them and men never get punished. They say that it is your manner and your appearance drove men to do so*.

The educational system was described by the participants as an important factor in accurately reacting to and confronting instances of harassing behavior. The participants believed that the education they had received at school and college had not provided them with any instructions concerning how to behave with men, how to confront harassing behaviors, or how to protect themselves.

*Since childhood, we [girls] have not received training in some areas, not at schools and not in the media. I always wonder why nobody taught us anything about our bodies, sexual matters, how to behave with the opposite sex, the potential of being harassed at any place, or how to react and protect ourselves in such situations*.

… *no one taught us anything … not on TV, not on the radio, not at school. It seems as if talking about this issue is a crime*.

A few participants stated that they had developed some personal methods based on their experiences. They added that they would suffer less psychological harm and abuse if they could have training on such matters. For instance

*We have learned what to do based on our experiences. I wish they had taught us such things, so that we might not suffer so much. We were raised as underdogs who would be dominated by any man.”*

### 3.4 Intervening conditions

The intervening, or mediating conditions of the current study were classified into two groups: individual factors and environmental factors. Each class was also divided into several subclasses. Individual factors included having audacity, and having financial needs, while environmental factors included the workplace atmosphere and policies, supervisors' personality traits, and emotional work. They are outlined in the following paragraphs.

Confrontational skills and audacity were among the characteristics that played important roles in managing transactions with customers who have harassing intentions. Some participants conceded that they lacked the required skills to confront harassing behaviors and that their inability caused them to become panicky or nervous.

… *girls are brought up as scared people. They have no courage to respond to men. Whenever they see harassing behaviors from men, they immediately start shaking and become panicky. I cannot respond to men. Whenever a man touches my hand, or gazes at me, I lose control. I have no such ability. I know responding is far better than staying silent, but of course, I cannot …*

Some participants emphasized that they had the ability to counteract harassing behaviors, but they could not actualize it due to the constraints of the workplace. For instance:

*I can respond well and I am not scared. However, I have gradually learned what type of response is more appropriate. In the past, I have seriously retaliated on many occasions, but we have limitations here that prevent us from behaving as we like … [even though] when we respond seriously, harassers stop what they are doing*.

Financial need was a major mediating factor that influenced participant's chosen response strategies. Ultimately, where there was a financial need to work, they had to remain silent against harassing behaviors to keep their job. As one participant stated:

*I really need my job. I* must *keep it to pay for my living costs. That I have to tolerate … whatever … is not pleasing. I have to remain silent in the presence of harassing customers, so that they may do their shopping*.

Environmental factors were considered another set of intervening conditions. Specifically, emotional work is a professional requirement for salespeople. The participants stated that saleswomen were supposed to show attractive behaviors toward customers in their workplace, to wear a smile, and act with cheerfulness. On the other hand, Iranian men tend to consider a woman's cheerful behaviors with other men as showing her desire to establish sexual relations, which can result in harassing and irritating behaviors against such women.

*wherever I have worked, my employer has told me to talk warmly and laugh with customers. I am supposed to start a conversation on any possible topic. Whenever a couple comes to the shop and the woman is in the fitting room, I am supposed to start a conversation with the husband, so that he may become tempted to buy something. They do not think about the acceptability of this behavior in our society. Those men might assume annoying things about me. Then, they start harassing me. They think that I am an indecent woman who wants to have a relationship with them*.

*our employer expects us to behave well in the workplace, so that we can sell a lot. We try to behave well with customers, so that they can choose comfortably, but they can easily get a wrong impression. They think we want to start a relationship as we speak with them cheerfully*.

The workplace atmosphere was also considered an intervening factor, particularly when neighboring shops shared facilities, which was a typical situation for small independent businesses. Participants mentioned that their workplaces did not provide safe spaces for women to move to. There was an atmosphere of almost constantly harassment, both by male customers and by salesmen in neighboring shops.

*Our workplace atmosphere is not good. I do not have the courage to leave the shop. Coming to the shop or leaving it is quite difficult for me because of the inappropriate comments and gazes of the salesmen in the neighboring shops. Sometimes they look so strangely at me that I become suspicious of myself. … This is an additional problem to being harassed by customers. As soon as I react to a harasser, everybody starts thinking inappropriate things about me. They blame me for such occurrences. We have to remain silent to maintain our honor*.

*The atmosphere here does not let me leave the shop as the neighboring salesmen or colleagues do not look appropriately. They start talking inappropriately. For example, they say the girl they saw was quite sexy. We have repeatedly asked to change the place of washing the brooms as men talk inappropriately; however, nobody listens to us. Recently, the situation has gotten worse, and they ignore a women's presence and intentionally use swear words to distress us. Because of their inappropriate behaviors, we do not dare to react when a customer starts harassing us. Our neighbors blame us! They say that we were responsible for the harassment, even when they have come to us and proposed to start a relationship*.

Employers' personality traits were also among the intervening factors that facilitated or constrained the participants' reactions against harassment. The majority of the participants expressed that most employers in the workplaces where they had worked were indecent men and sexually harassed their saleswomen. They also asked the women to behave quite openly toward customers.

*In most places where I have worked, my employers were not decent men. One of them proposed a “Sigheh” marriage to me (The term refers to a religious tradition in Iran that allows a temporary sexual relationship for several days; however, the public views it as profoundly unacceptable and undesirable and equates it with prostitution). Some of them proposed to start a relationship. I quickly gave up those jobs. Many employers are like that; they harass their saleswomen. Although my current employer has not proposed such things, he asks me to flirt with other men. If he was a decent man, he would never ask me to behave like that. They are all the same, though he is much better than many other employers. Decent men never employ female salespeople to sell men's clothes or home appliances; they employ men*.

Another important intervening factor was workplace policies that prevented the participants from reacting against harassing behaviors. The participants mentioned that one of their employment conditions was to behave openly and cheerfully with customers as well as to wear attractive clothes and makeup. Whenever a saleswoman reacted to a harassing customer or wore simple clothes, she would lose her job.

*Many employers request our photos before employment. They want to see if we are pretty or not. Many of them just employ pretty girls. If you are a modest girl, they will tell you right from the beginning that you have to change your clothes and appearance and attract customers. All private workplaces are like that. Everybody just thinks about their benefits*.

### 3.5 Strategies to stop harassing behaviors

The selection of different strategies would bring about certain consequences. A spectrum of strategies and consequences were identified in the current study. The participants' strategies against sexual harassment were classified into three categories, namely silence, avoidance, and counteracting. They often remained silent against sexually harassing behaviors because of the consequences of any counteraction. Their experiences showed that reacting to customers' harassing behaviors would lead to job loss. However, they said that their reactions varied depending on the type of harassment. Whenever they were sure that their employers were not present in the workplace, they warned the harassing customers or retaliated. Instances of the participants' statements have been presented below:

*Typically, whenever somebody intends to harass me, I say that I am sorry for him and do not start a quarrel as I want to keep my job. I usually remain silent and do not argue with them as they may […]. When they gaze or comment inappropriately, I do not say anything, but when they become aggressive and try to touch my body, I leave the shop …*.

*I typically remain silent, as my employer who is sitting there does not know that a customer is touching my body. If I scream, four other customers will leave the shop. I repeatedly say please check the camera, so that you can see he was touching me, but my employer says that I should remain silent, so that other customers may not leave the shop. They ask me to be silent and say nothing. This is so crazy. Whenever they comment inappropriately, I try to keep calm, but sometimes they feel my body. On those occasions, I react to the customer if I know that my employer will not come to the shop on that day. But when he is there, I just have to move away from the counter*.

### 3.6 Consequences

The consequences were divided into four classes in the current study: psychological impacts, job loss, family challenges, and putting one's marriage at risk. The psychological impact of facing harassment and counteracting harassers had a strong, negative impact on their mental health. Participants reported that they experienced feelings like sadness, guilt, fretfulness, mental involvement, nervousness, depression, stress, hatred, insecurity, and the lack of any connection to the world after facing harassing behaviors.

*Whenever I see such behaviors without being able to do anything, I feel very bad. I hate everything. I keep to myself. Sometimes, I even start crying. It is then on my mind all day*.

*In such situations, I feel that I do not belong to this world. I am a girl who wants to live freely and safely. When I do not want to be harassed, they should not approach me. It is obvious that a girl does not want men to gaze indecently at her or touch her. Such occurrences cause us to become depressed*.

Losing one's job due to counteracting a harassing customer was the second consequence. Many participants said that they remained silent in the face of harassing behaviors, as they feared losing their jobs. Their experiences showed that reacting to customers' harassing behaviors would result in employers' anger, being scolded by them, job loss, and necessity to look for another job opportunity.

*I have many years of experience in this job; however, I was fired by my employers when I counteracted harassing customers*.

*When I have reacted [to harassment], my employer becomes angry. He says that my intention is to scare customers. I have lost my job because of this once or twice. Then I was forced to look for another job. Then, I understood that reacting to a customer was so costly*.

The third consequence was the family challenges that arose because of facing and reacting to harassing behaviors on the part of male customers. The participants mentioned that facing improper behaviors in their workplaces disturbed their moods, which resulted in arguments inside their families. For example:

*Customers' harassing behaviors cause arguments at home, as I sometimes tell my husband that if he could supply the household, I would not be forced to work in such places or face such behaviors. Sometimes, when family members get informed of such occurrences, they force us to change our workplaces or do not let us find another job. Family members start to quarrel. Some of them come to check the situation or ask questions*.

*Facing such behaviors obviously affects one's mood. It stays on your mind. It spoils the whole day. When you go home with that disturbed mind, you unintentionally start quarreling with your family members because of simple matters because you have become disturbed at your workplace*.

Putting one's marriage at risk was the fourth consequence for the saleswomen. The participants stated that constantly facing indecent behaviors on the part of male customers (particularly married men) made them lose trust in men. They also pointed out that such distrust caused them to feel profound and serious doubts about marriage and to become unable to choose a partner for life. For instance,

*I completely distrust men. I have always seen indecent behaviors by men in my workplace, and this has caused me to distrust them. I even cannot choose a man to marry. I have lost my trust in men since I was 27 years old. It causes me to keep my distance. I feel that all men are the same. There is not a single good man*.

*Such behaviors prevent me from trusting other men. Sometimes, I become scared and my hands and legs start shaking. I am scared of marriage … I feel that no man is good, that all of them are indecent. I always feel unsafe. Whenever a man comes to our house for the proposal ceremony, I think that he is one of the indecent ones. I cannot choose a man*.

### 3.7 Storyline

**Figure 1:**
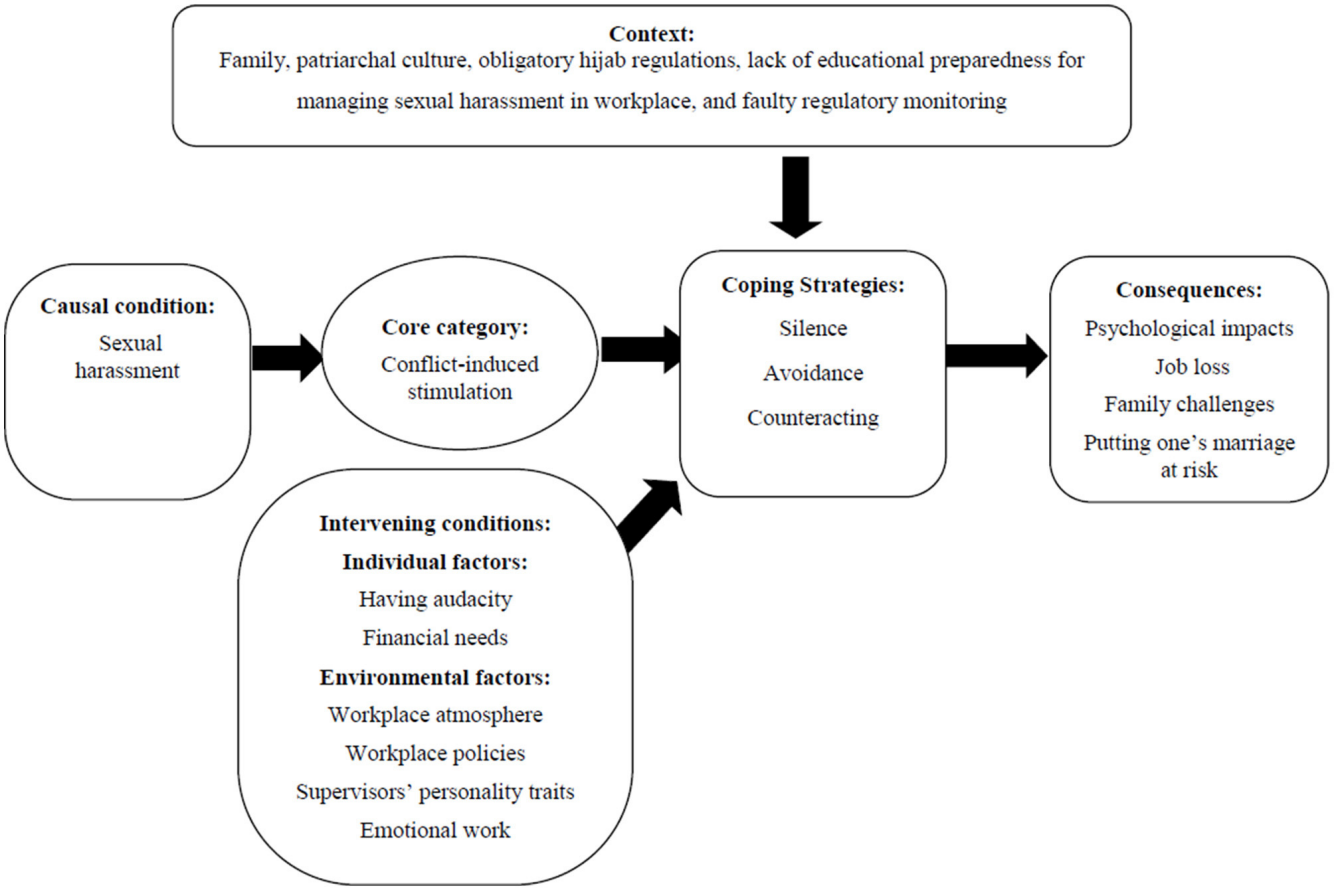
Grounded theory model identifying causal conditions, core category, context, intervening conditions, strategies and consequences for retail saleswomen in Shiraz confronted with sexual harassment at work.

People suffer conflict-induced stimulation when they face the stimuli of harassing behaviors. Stimulation means the readiness or tendency to react, while conflict arises in the form of doubts when deciding and selecting what reaction to make. In the present study, the participants adopted the following confrontation response strategies: silence, avoidance, and counteraction. Depending on the type of the selected strategies and other factors, a spectrum of different consequences—including psychological impacts, losing one's job, family challenges, and putting one's marriage at risk—could arise. Additionally, two classes of factors including intervening conditions (individual and environmental factors) and contextual conditions (such as the characteristics of the society, family mores, patriarchal culture, obligatory hijab regulations, lack of education about sexual harassment in the workplace, and faulty legal monitoring mechanisms) influence the process of counteraction and its consequences. Moreover, the expectations of the workplace employers (intervening conditions) were different from those of employees' families and the society (contextual factors) provoking conflict. While family and society dictate one set of behavioral frameworks, workplaces prescribe different frameworks. These opposing forces cause tension in people while deciding how to behave in the face of harassing behaviors (stimulus or causal conditions), which could, in turn, bring about a number of consequences (see Figure 1).

## 4 Discussion

Sexual harassment is a prevalent phenomenon with unknown dimensions and angles which are influenced by contextual conditions and characteristics of societies. The current study investigated the problem in women working in retail clothes shops in Iran. Key objectives were to get a better understanding of the experience of sexual harassment in this group and particularly the *how* and *why* of their responses when confronted by sexual harassment. This knowledge is required to generate theories of the underlying reasons for the prevalence of the problem that can be tested, and in due course to develop appropriate interventions to protect retail sales women from the distress of sexual harassment. The findings that emerged from the in-depth unstructured interviews were that the main perpetrators of sexual harassment were male customers, and that response strategies could be put into three broad categories—silence, counteracting, and avoidance. We also found that the most prevalent response was to suppress any motivation to react and to stay silent. Taking full account of the participants' experiences—a judicious selection is given in this manuscript—we interpret remaining silence as an active coping strategy. Remaining silent is a choice made in the context of powerlessness in a system of patriarchal control as a means of reducing any further distress. Remaining silent is an active response just as much as counteracting and removing oneself to avoid confrontation. Altogether, the findings presented a complex picture of motivated strategies used in a response to sexual harassment made up of interconnected and layered nexuses.

A key driver for response strategy used was the recognized consequences. Despite the distress of sexual harassment, the personal cost of counteracting was often perceived as being likely to cause even more distress. All participants acknowledged that their experiences of sexual harassment were that these situations led to physiological and psychological stimulation. That is, by definition sexual harassment is a stressor that precipitates a state of bodily and mental readiness to respond as a matter of urgency (Chrousos, 1998). Nevertheless, in the situation of sexual harassment participants were faced with conflict and a need to decide or choose between action options to minimize all potentials for stress. Various factors were found to influence their decision-making. These are discussed below as “contextual” and “intervening” conditions.

Contextual conditions included the characteristics of the Iranian society—the patriarchal culture, the central important of family, obligatory hijab regulations, the characteristics of the education system, and legal monitoring. The educational system should be recognized as being significant in terms of being able to provide the important preparation girls and young women need for managing potential sexual harassment when they enter the world of work. Educational input could include familiarization with the main preventative behavioral models and how they can be applied in roles undertaken by females. Educational input in the schooling system could provide the skills female employees need to appropriately manage sexual harassment and the expectations of their future work role by employers and family members. Participants recognized that this form of support was a significant gap in their education. Educational input about sexual harassment would be beneficial in all countries—especially those with a patriarchal culture.

Most of the different types of sexual harassment reported in this study—verbal, gestural, physical, and psychological—have been identified in the previous studies. However, harassment in the form of men proposing a Sigheh marriage to female sales assistants was found as something new. Sigheh is a religious principle in Islam and refers to a temporary matrimonial relationship. Most of the Iranian society does not approve of establishing such relationships (Ahmadi et al., 2012), and the payment of a dowry involved is likened to legalizing prostitution. Consequently, proposals for a Sigheh marriage are regarded as a type of sexual harassment. Muslim families expect female members to behave solemnly with men in the workplace, not to encourage interactions and to have approval rights for relationships. These extend to pressures to observe a standard dress code dates (Zahed and Kaveh, 2013). Iranian society regards the chador as the symbol of dignity and high status for a woman, and women who do not cover themselves completely, move away from the definition of good women, and are more vulnerable to sexual harassment (Lahsaeizadeh and Yousefinejad, 2012). In Iran, there are also traditional religious views which regard women as tempting creatures, and attribute all faulty behaviors to them (Riahi and Lofti Khachaki, 2016). Such views are another source of motivation for harassing parties. In this cultural context, women become disarmed against harassing behaviors and experience conflicting feelings as they consider their reactions useless.

Intervening conditions were influential in the emergence and intensification of conflicts, and in the selection of strategies. Individual factors were among the most important intervening conditions that influenced conflict-induced stimulation. Bishop Mills and Scudder (2023) argued that the most effective reaction to sexual harassment was a vigorous response that supported the positive image of the harassed women, while avoidance was found to have the least impact. Thus, stringency and vigorous-empathetic responses were perceived as effective strategies in decreasing the rates of harassment in future. The findings of the current study also showed that audacity played a critical role in successfully managing interactions with customers with harassing intentions. However, overall, whilst some women could counteract harassment, others lacked such capabilities. This result was in line with other studies which have reported that women usually adopted passive strategies against harassing behaviors (Adams et al., 2019), although as alluded to previous, Chubin (2014) pointed out that women remain silent against harassing behaviors but this is purposeful, not passive. Non-combative reactions can also be attributed to cultural-organizational factors and participants' endeavors to manage the pressure of professional relationships (Gruber and Smith, 1995). Prior studies which have investigated the effects of power relations within organizations on the occurrence of sexual harassment have found a woman's status to be remarkably influential (Loy and Stewart, 1984). When the victims of sexual harassment were in the higher ranks of an organization, stronger reactions were made (Perry et al., 1997). Furthermore, when employees perceived that their workplace atmosphere supported them against sexual harassment, they responded more strongly (Bingham and Scherer, 1993). This was agreement to these the findings of the current study, insofar as the workplace atmosphere did not support women against sexual harassment, and the people in workplaces sometimes became the harassing party, hence sexual harassment prevailed.

In terms of the emotional nature of work, smiling, empathizing, and keeping calm, are among the most significant requirements of the service industry to maintain customers and to support best performance of organizations (Grandey, 2000). In other words, sexual attraction and flirting are often considered a part of an employee's professional tasks (Yagil, 2008). Nevertheless, when such emotional behaviors are conducted by saleswomen to attract customers, there is much scope for misunderstandings. Critically, the unclear boundary between professional and personal lives can result in sexual harassment in workplaces (Good and Cooper, 2016). In this study participants confirmed that they were expected to ignore sexual harassment. Thus, to ignore flirtatious customers or to protest about their harassing behaviors would be regarded as a violation of workplace norms. Moreover, as previously reported by Yagil (2008), explicitly confronting a misbehaving customer could bring about negative consequences. Such situations resulted in the emergence of cognitive conflicts amongst the women. To some extent such situations in workplaces arise due to faulty legal mechanisms. The introduction of regulations and reforms in the society is beneficial in raising people's awareness of sexual harassment and forming principled norms in workplaces (McDonald, 2012). However, the absence of supportive systems to report sexual harassment can lead to and intensify harassment and its destructive impacts on women's health. Rostamzadeh and Mehregan (2016) indicated that there were no laws in Iran to support women against workplace sexual harassment. This is an organizational issues across the globe and historically, courts of law have not presented a positive attitude toward supporting women who report sexual harassment and even adopted a non-empathetic perspective in such instances (Fitzgerald et al., 1995). Critically, as women feel that the courts of law will not uphold their evidence-based claims, they cannot imagine a positive outcome for strategies that include confronting the harasser, or reporting such incidents (Knapp et al., 1997). A recommendation from this research, which may not be new, but clearly needs to be repeated, is that legal and monitoring contexts need to be available so that women are not conflicted in terms of how to confront and counteract in such situations, and they can be safe in their employment. That is, systems should be in place in every workplace, large or small, to effectively tackle harassment at source (Cousins et al., 2004). It really is an injustice to use knowledge that employees have financial needs and are dependent on their jobs, to turn a blind eye to sexual harassment, and perhaps even more so when an employee who counteracts harassment can be punished by losing their job or reducing their financial benefits in some other way (Knapp et al., 1997).

The present study confirmed previous findings of the impact of sexual harassment persist in Iran, including job stress, reduced job satisfaction, lower organizational performance, quitting one's job, and physical/mental illnesses, and poor psychological wellbeing (e.g., Glomb et al., 1997; Willness et al., 2007; Dionisi et al., 2012). It is [still] difficult for women to draw upon the notional support of the law, and the business case for doing so is poorly understood. The findings of the current investigation additionally demonstrated that becoming a victim of workplace sexual harassment brought about certain psychological changes in people's mood and behaviors, which influenced their family life. Participant's interview data revealed that sexual harassment initially led to behavioral changes such as social withdrawal, keeping to themselves and spending all their energy on their experience of harassment, which went on to harm or even destroy their relationships in their families. In this regard, Zhu et al. (2021) emphasized that customers' boldness influenced the emergence of conflicts between workplaces and families and could weaken the foundations of families. An interesting gap in the findings was a preference for advocacy seeking for support—which one would expect from an employer or manager on the basis that employees are huge assets to the business (Mackay et al., 2004). Zhao and Ghiselli (2016) suggest that in the context of sexual harassement, women need to implement every available strategy to maintain their jobs, but they become more sensitive to the tiniest changes in their workplaces and families, and this leads to a high rate of workplace-family conflicts, eventually resulting in higher stress levels for the worker. Aligned to this point, another consequence of facing sexual harassment was the formation of distrust and pessimism against all men among many of the saleswomen. This attitude made it remarkably difficult for them to go forwards with the normal development of relationships toward choosing a life partner and marriage. We found no similar study reporting this finding, and we suggest that this mental barrier again all men based on the negative experiences from a subset of men calls for further investigation.

### 4.1 Strengths and limitations

The current study was pioneering in terms of adopting a behavioral grounded theory approach to explore sexual harassment, and propose a storyline to explain the process of such occurrences. The storyline provides an explanation of the phenomenon of sexual harassment in this population which can be tested. A key limitation is that the current study was conducted on a Muslim population in a Muslim society. This is a very large population and not limited to Iran, however, the findings may not be generalized to female employees who belong to other communities.

## 5 Conclusion

Sexual harassment induces conflict-induced stimulation and responses are influenced by intervening conditions, contextual factors, selected strategies, and the perceived consequences of the response. The findings of the grounded theory study suggest that there are negative consequences, particularly in terms of lack of employer support and losing one's job, shame, and family disapproval which act as barriers for female saleswomen to counteracting sexual harassment from male customers. Such an understanding can be applied to develop educational policies to support women and ameliorate the prevalence of this essentially illegal problem. The grounded theory approach produced a storyline that can be tested. Overall, the findings can be generalized to Middle Eastern societies that are similar to the Iranian society from a religious perspective.

## Data availability statement

The raw data supporting the conclusions of this article will be made available by the authors, without undue reservation.

## Ethics statement

The studies involving humans were approved by the Ethics Committee of Shiraz University of Medical Sciences, Shiraz, Iran (IR.SUMS.REC.1398.1366). The studies were conducted in accordance with the local legislation and institutional requirements. The participants provided their written informed consent to participate in this study. Written informed consent was obtained from the individual(s) for the publication of any potentially identifiable images or data included in this article.

## Author contributions

MA: Data curation, Methodology, Project administration, Writing – original draft. MK: Conceptualization, Supervision, Writing – review & editing. RC: Supervision, Writing – review & editing.
